# Serotonergic Signaling Rewired: A Lipid Raft-Controlled Model of Synaptic Transmission Grounded in the Fundamental Parameters of Biological Systems

**DOI:** 10.3390/life16010118

**Published:** 2026-01-13

**Authors:** Jacques Fantini, Marine Lefebvre, Nouara Yahi, Henri Chahinian

**Affiliations:** 1 Department of Biology, Faculty of Medicine, University of Aix-Marseille, INSERM UA16, 13015 Marseille, France; nouara.yahi@univ-amu.fr (N.Y.);; 2 IHU Méditerranée Infection, 19-21 Boulevard Jean Moulin, 13005 Marseille, France; marine.lefebvre.1@etu.univ-amu.fr; 3 Microbes Evolution Phylogeny and Infections (MEPHI), Aix-Marseille Université, 27 Boulevard Jean Moulin, 13005 Marseille, France

**Keywords:** serotonin, cholesterol, ganglioside, synapse, solubility, plasma membrane, fundamental parameters, quantum biology, electrostatics

## Abstract

Serotonergic signaling is traditionally conceived as a transient, vesicle-mediated process restricted to the synaptic cleft. Here, we propose an expanded model in which serotonin can also be inserted into the plasma membrane of neurons and glial cells, forming a stable, membrane-associated reservoir that prolongs its availability beyond classical synaptic timescales. In this framework, the synapse emerges not as a simple neurotransmitter–receptor interface but as a dynamic, multiscale medium where membrane order, hydration, and quantum-level processes jointly govern information flow. Two temporal “tunnels” appear to regulate serotonin bioavailability: its aggregation in synaptic vesicles during exocytosis, and its cholesterol-dependent insertion into neuronal and glial membranes at the tripartite synapse. Lipid raft microdomains enriched in cholesterol and gangliosides thus act as active regulators of a continuum between transient and constitutive serotonin signaling. This extended serotonergic persistence prompts a reconsideration of current pharmacological models and the action of antidepressants such as fluoxetine, which not only inhibits the serotonin transporter (SERT) but also accumulates in lipid rafts, perturbs raft organization, and alters serotonin–cholesterol equilibria, contributing to SERT-independent effects. Grounded in the recently established fundamental parameters of biological systems, this model invites a broader, quantum-informed rethinking of synaptic transmission.

## 1. Introduction

**Figure 1:**
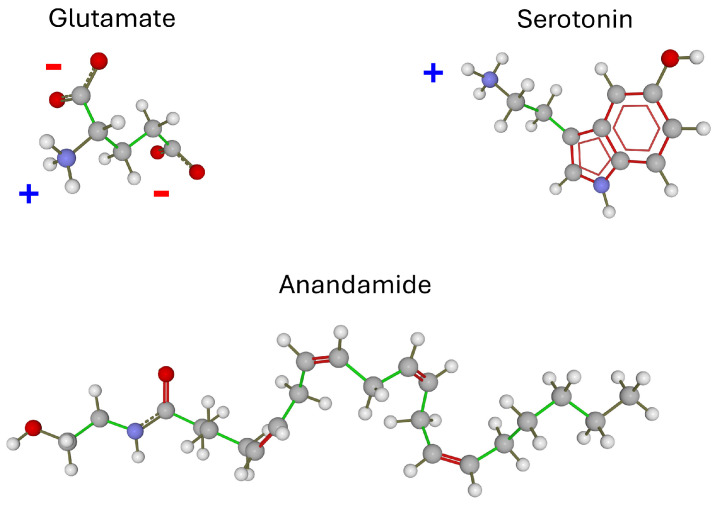
**Chemical structure of glutamate, serotonin and anandamide.** At pH 7, the net charge of glutamate is −1, that of serotonin is +1, and anandamide is neutral. These three neurotransmitters differ significantly in their water solubility, in descending order of glutamate > serotonin > anandamide. Oxygen atoms are red, nitrogen blue, carbon grey, and hydrogen white. Chemical bonds are colored differently (green, grey or red) for clarity.

In the classic view of synaptic neurotransmission, it is usual to associate a neurotransmitter with its membrane protein receptor as a functional duet [1]. Although this dual entity is generally presented as sufficient to describe the mechanisms of chemical neurotransmission, auxiliary parameters are also necessary [2,3]. For instance, plasma membrane microdomains such as lipids rafts, in which cholesterol and sphingolipids are concentrated [4], exert a chaperone effect on neurotransmitter receptors conformation [5,6,7], highlighting the epigenetic dimension of membrane receptor structure [8,9,10]. On one hand, numerous studies have identified cholesterol as a major regulator of acetylcholine and serotonin receptors activity [11,12]. On the other hand, sphingolipids, in particular gangliosides [13], appeared as important partners of neurotransmitter receptor structure [14,15,16]. Both lipids have also been shown to interact with serotonin [12,17,18,19,20,21]. This challenges the centripetal way of thinking that neurotransmitter targets are exclusively proteins in nature. In this review, we analyzed recent data demonstrating that cholesterol and gangliosides take part directly or indirectly in the dual receptor model of neurotransmission. We focused our attention on three neurotransmitters with marked differences in water solubility: anandamide, glutamate and serotonin (Figure 1). Then we present a lipid raft-based model of serotonergic transmission which challenges the classic dogma of synaptic communication. Building on synaptoneurolipidomics, which details lipid compositions at junctions and tools to probe their synaptic roles [22], our model integrates cholesterol-ganglioside synergies specific to serotonergic rafts.

## 2. Auxiliary Role of Gangliosides in Synaptic Clearance of Glutamate

An unexpected function of gangliosides in glutamatergic neurotransmission has been recently deciphered [23]. This discovery is based on the understanding of the excitotoxic properties of this anionic neurotransmitter (Figure 1), which must neither enter the postsynaptic neuron nor remain for long periods in the synaptic cleft [24,25]. Excess glutamate is rapidly transported to the third partner in the tripartite synapse, the astrocyte [26]. While this mechanism had been well established for many years, an explanation was lacking to understand why glutamate molecules are selectively directed towards the astrocyte rather than towards pre- and postsynaptic neurons. This is where the gangliosides of the rafts come into play. Biochemical analyses of the ganglioside composition of the three cells of the tripartite synapse have shown a strong asymmetry in expression. The pre- and postsynaptic neurons are rich in large and highly charged gangliosides (GM1, GD1a, GD1b, GT1b), while the astrocyte possesses only one type of ganglioside, GM3 [27]. This monosialylated ganglioside has only one negative charge, and its glycan polar region is smaller than that of the gangliosides in neurons. Thus, the electric field generated by the rafts of the pre- and postsynaptic neurons is much stronger than that of astrocytes, creating a permanent flow carrying glutamate molecules towards the astrocyte [23].

**Figure 2:**
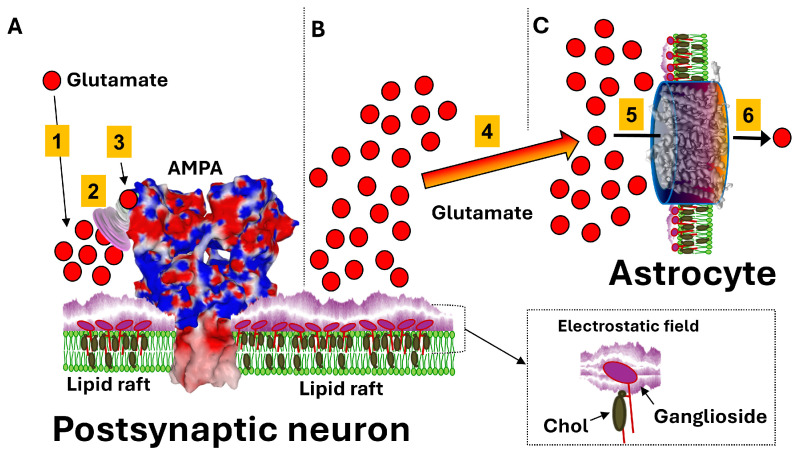
**Schematic representation of the glutamatergic synapse.** The figure represents three distinct periods of synaptic glutamate transmission: (**A**) glutamate binding to post-synaptic receptors, (**B**) neuron-astrocyte flux of glutamate controlled by electrostatics, (**C**) glutamate transport into astrocytes. After synaptic release, glutamate molecules (red disks) diffuse within the synaptic cleft and reach post-synaptic receptors (e.g., α-amino-3-hydroxy-5-methyl-4-isoxazolepropionic acid AMPA glutamate agonist receptor) concentrated in lipid raft microdomains (steps 1–3). The negatively charged sialic acids of post-synaptic gangliosides create a surface electrostatic potential that repels the anionic glutamate molecules from the neuronal membrane. In contrast, the AMPA receptor presents a positively polarized “electrostatic funnel” (step 2) that attracts glutamate and steers it toward the orthosteric binding pocket (step 3). The distribution of basic residues and the overall shape of the receptor’s extracellular domain focus electric field lines so that approaching glutamate molecules are guided into the ligand-binding cleft, increasing the probability and speed of productive binding despite the global repulsive field generated by adjacent gangliosides. Excess extracellular glutamate diffuses away from the synapse (step 4) and is cleared by glutamate transporters (EAATs) localized in the plasma membrane of adjacent astrocyte (step 5). Astrocytic uptake limits excitotoxic accumulation of glutamate and contributes to neurotransmitter recycling (step 6). As discussed in the text, most of these mechanisms (including the electrostatic funneling of glutamate) have been experimentally validated. The concept of an electrostatic field generated by neuronal raft gangliosides driving glutamate flux to astrocytes remains, at this stage, a theoretical framework grounded in established biophysical principles [23]. Inset: in lipid rafts, gangliosides are associated with cholesterol (chol).

Brain gangliosides act therefore as auxiliary factors helping the neurotransmitter to find its way to the post-synaptic membrane, thereby speeding up the whole process of synaptic transmission. The differential expression of gangliosides in the three cellular partners of the tripartite synapse drives the clearance and recycling of excess glutamate (Figure 2), which is irreversibly directed toward the astrocyte and then recycled through a glutamate–glutamine cycle [28,29]. The price to pay for such a system to function is the enormous size of the postsynaptic glutamate receptors, whether ionotropic or metabotropic [23]. Thus, the glutamate binding site is outside the influence of the repulsive electrostatic field generated by the gangliosides of the postsynaptic neuron. A vortex created by an electrostatic funnel at the distal end of the receptor also promotes glutamate binding to its site. This provides a perfect example of the importance of electrostatic forces in the synaptic transmission mechanism of glutamate. These forces are generated and controlled by the gangliosides of the rafts, which are therefore essential partners in the functioning of the synaptic machinery.

This model, initially based on molecular dynamics simulations, has been validated by site-directed mutagenesis experiments [30]. Free energy calculations identified specific positively charged surface residues that formed a metastable pathway (funnel) to guide glutamate into the binding pocket. The authors of this study created mutant receptors where these charged residues were neutralized (alanine mutants) to disrupt the electrostatic steering. The mutants showed significantly slower activation rise times (3-fold slower) and deactivation rates compared to wild-type, even though the equilibrium affinity was largely unchanged. This experimentally confirmed that the funnel is responsible for the speed of binding, not just the final binding strength, in agreement with the proposed role of electrostatic fields in synaptic transmission [23].

## 3. Cholesterol as a Functional Co-Receptor Controlling the Retrograde Synapse

At the other end of neurotransmitters water solubility scale, endocannabinoids such as anandamide are probably the neurotransmitters that are the most concerned with lipid interactions because they are lipid themselves. However, as for glutamate, the endocannabinoid neurotransmission is generally presented as a standalone neurotransmitter-receptor story, without any mention to lipid rafts or even to membrane lipids.

**Figure 3:**
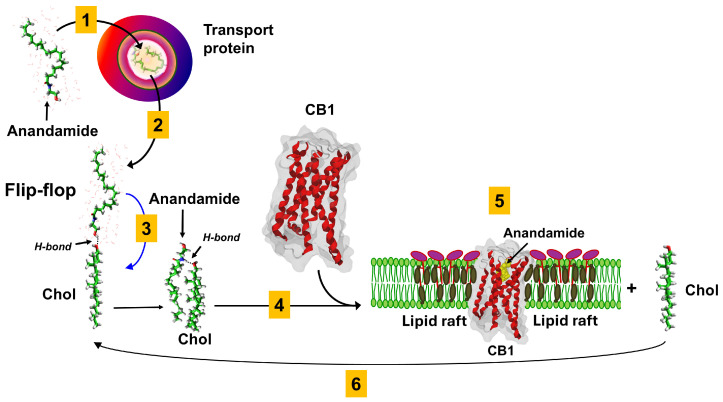
**Membrane trafficking and receptor targeting of anandamide at CB1 receptors in lipid rafts.** Anandamide is synthesized in the postsynaptic neuron, released into the synaptic cleft, and then travels retrogradely to activate CB1 receptors located on the presynaptic terminal. During this journey it is not freely diffusing in bulk water but is thought to be escorted and/or shielded by carrier mechanisms, including specific transport proteins and lipid-based vehicles, which effectively “vehicle” anandamide from the postsynaptic to the presynaptic side (step 1). After initial binding to cholesterol (chol) in the post-synaptic membrane (step 2), anandamide inserts into the membrane by a flip-flop like mechanism (blue arrow), which facilitates its subsequent lateral diffusion within the membrane (step 3). Anandamide then partitions into lipid raft microdomains where it reaches CB1 receptors, with cholesterol acting as a molecular chaperone that stabilizes receptor conformation and ligand binding (step 4). Within the CB1–cholesterol–anandamide complex, cholesterol can dissociate, allowing receptor deactivation or recycling (step 5). The overall cycle illustrates how cholesterol-dependent lipid raft organization controls anandamide delivery, CB1 activation, and subsequent clearance from the membrane (step 6). These molecular mechanisms, including the specific cholesterol-assisted insertion and translocation of anandamide across the lipid bilayer, have been experimentally validated using reconstituted membrane models [31,32].

However, it is impossible to disconnect the retrograde synapse of endocannabinoids from membrane lipids. Thus, our team highlighted the predominant role of cholesterol in the membrane insertion of anandamide [31,32]. This step is crucial for neurotransmission because the binding site of anandamide on the cannabinoid receptor 1 (CB1) is not accessible from outside the membrane [33,34]. Located within the transmembrane domains of the receptor, this site is completely immersed in the most nonpolar regions of the lipid bilayer [35,36]. Anandamide must therefore first insert itself into the bilayer, then migrate within the membrane until it reaches CB1 (Figure 3). From a kinetic point of view, this mechanism is very slow, and it would be even slower if it relied entirely on stochastic events. This is not the case, thanks to the action of cholesterol, which plays a dual role in this particular type of synapse: (i) it attracts anandamide and (ii) detaches it from its transport protein, ensuring the shuttle between the transmitting neuron (postsynaptic in this case) and the receiving neuron (presynaptic) [37]. This transport protein plays the same role as serum albumin for the transport of fatty acids [38], allowing lipids to diffuse in an aqueous environment [39].

We have suggested that cholesterol could be both considered as a membrane co-receptor and transporter of anandamide (AEA) or monoacyl-glycerol (MAG) in the membrane plan [31]. The complex endocannabinoid-cholesterol might diffuse through the membrane plane to target cannabinoid membrane receptors but also a cholesterol molecule located in the inner leaflet of membrane which will allow its translocation by diffusion through the membrane [31]. A fatty acid binding protein allowing the intracellular transport of endocannabinoids is also involved in the intracellular clearing process of retrograde synapse allotted to presynaptic neurons [40] which ends by arachidonic acid production according a FAAH dependent endocannabinoids hydrolysis [41].

**Figure 4:**
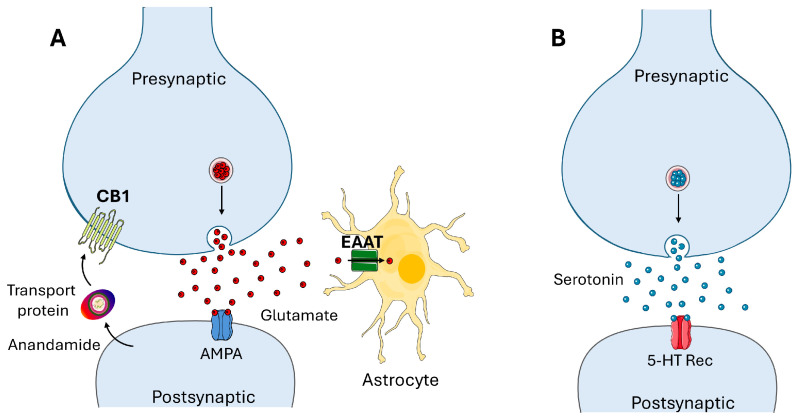
**Synaptic Transmission Mechanisms at Glutamatergic and Serotonergic Synapses** (**A**). Schematic representation of a glutamatergic synapse showing presynaptic glutamate release and postsynaptic AMPA receptor activation, with surrounding astrocyte expressing excitatory amino acid transporter (EAAT) to clear extracellular glutamate; the retrograde activity of anandamide released by the postsynaptic neuron and delivered to the presynaptic membrane receptor CB1 by a transport protein is also schematically depicted. (**B**)**.** Classic representation of a serotonergic synapse showing presynaptic serotonin release into the synaptic cleft and subsequent activation of postsynaptic serotonin (5-hydroxytryptamine, 5-HT) receptors. Images provided by Servier Medical Art (https://smart.servier.com), licensed under CC BY 4.0 (https://creativecommons.org/licenses/by/4.0/, accessed on 1 January 2020).

From a physiological point of view, anandamide exerts a retrograde control that dampens glutamatergic synaptic activity by limiting presynaptic glutamate release. This negative feedback “calms down” the excitatory synapse and helps prevent excessive postsynaptic AMPA receptor stimulation (Figure 4A).

## 4. Role of the Ganglioside/Cholesterol Duet in the Clearance of Serotonergic Synapse

**Figure 5:**
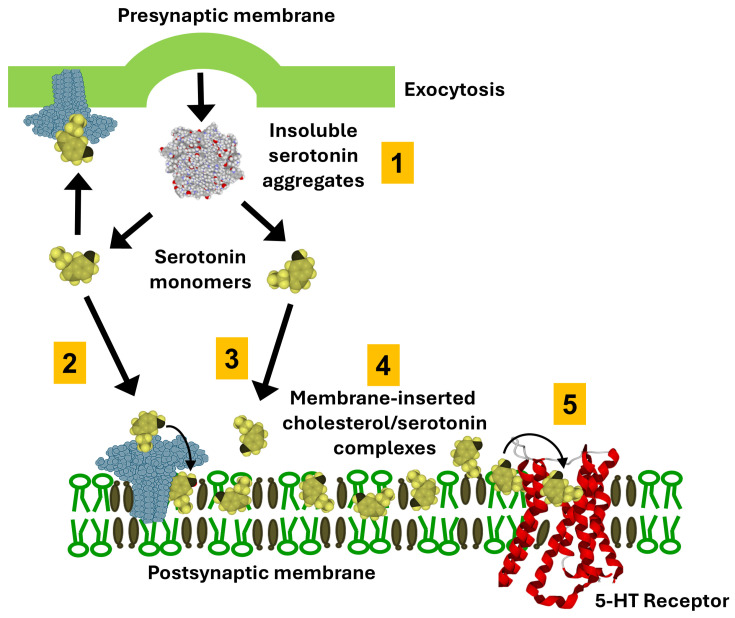
**Ganglioside/Cholesterol-mediated handling of serotonin at the postsynaptic membrane.** Following exocytotic release, serotonin aggregates in the extracellular space (step 1). Serotonin monomers are attracted at the post-synaptic membrane by gangliosides (step 2) and cholesterol (step 3). Gangliosides are colored blue, cholesterol brown, and phosphatidylcholine green. A fraction of monomeric serotonin partitions into the postsynaptic membrane where it forms mixed cholesterol/serotonin complexes (step 4) that accumulate within lipid raft microdomains enriched in the ganglioside GM1. Within these rafts, GM1 interacts with the indole ring of serotonin through CH–π and electrostatic contacts (inset), thereby stabilizing monomeric serotonin at the membrane surface and facilitating its presentation to nearby 5-HT receptors (step 5). Membrane-inserted cholesterol/serotonin complexes may undergo recycling, contributing to local buffering of serotonin concentration and modulation of 5-HT receptor activation dynamics. The specific interaction of serotonin with cholesterol and gangliosides has been demonstrated using reconstituted membrane models (Langmuir monolayers and solid-state NMR) [43,55]. The overall mechanism remains, at this stage, a theoretical framework grounded in established biophysical principles.

Serotonin signaling at the synapses classically relies on the tight duet between the neurotransmitter and its receptors (Figure 4B). After an action potential invades the serotonergic terminal, serotonin is released into the synaptic cleft and binds postsynaptic 5-HT receptors, which convert this chemical signal into intracellular responses that ultimately shape neuronal excitability and network activity. This classic “neurotransmitter–receptor duet” description is indeed incomplete because it treats serotonin as if it were just a freely diffusing, homogeneous solute in the cleft. In reality, serotonin’s physicochemical properties—such as its protonation state, moderate water solubility, and capacity for electrostatic and cation–π interactions with membrane lipids and proteins—strongly influence its local concentration profile and receptor access. In fact, serotonin is amphipathic and is released from synaptic vesicles at concentrations that exceed its aqueous solubility limit (approximately 270 mM in vesicles versus 110 mM solubility). Consequently, serotonin predominantly exists as aggregates in the synaptic cleft, driven by extensive aromatic stacking interactions (Figure 5). The value of 110 mM is independently supported by Nag et al. [42], who demonstrated through osmometry and NMR that serotonin remains monomeric only up to 100–150 mM, above which extensive aggregation occurs through π-stacking. Their biophysical measurements, using completely independent methodology, converge with our experimental findings [43]. It should also be noted that commercial specifications listing serotonin solubility refer to serotonin hydrochloride, not the natural free base form. The field has systematically used salt forms for experimental convenience, leaving fundamental physical properties of natural serotonin uncharacterized. Until 2022, even the crystal structure of free base serotonin had never been reported [44]. Interestingly, similar issues exist for other aromatic biomolecules such as adenine [45]. Adenine undergoes base-stacking interactions (π–π stacking) and can form aggregates that are reportedly dispersed by certain sugars, such as galactose or glucose [46].

The aggregation of serotonin has been largely overlooked, as conventional models often assume serotonin is monomeric following vesicle exocytosis. Employing a combination of in silico simulations, physicochemical assays, and a novel experimental system mimicking synaptic conditions, we recently demonstrated that serotonin aggregates are effectively solubilized by gangliosides [43], particularly GM1 found in postsynaptic neuronal membranes, whereas GT1b, abundant in presynaptic membranes, lacks this effect [47]. This solubilization process, mediated primarily by electrostatic interactions, facilitates the dissociation of insoluble serotonin micelles into monomers. The serotonin-ganglioside interaction is mediated by a combination of CH-π [48,49,50,51] and electrostatic interactions [52] acting in synergy. Additionally, serotonin binds strongly to cholesterol through CH-π and van der Waals interactions [53], promoting retention of monomers within the membrane. Together, gangliosides and cholesterol are likely to function synergistically as a serotonin-collecting funnel on neuronal membranes. This model highlights a novel mechanism of serotonergic transmission that incorporates the post-exocytotic solubilizing influence of membrane gangliosides and cholesterol on serotonin aggregates.

An asymmetric distribution of gangliosides in the synapse might induce a polarization of the serotonin flow from the presynaptic to the post-synaptic neuron. However, this tropism could be modulated by the occurrence of an active metabolism of sialic acid moiety at the synaptosome membranes level catalyzed by neuraminidase and siallyltransferase [54]. These enzymatic activities modulate the permanent presence of GT1b at the presynaptic neuron membranes level. In that case the collecting funnel capacity of serotonin by pre-synaptic membranes cholesterol must be taken into consideration and might explain the retro-control activity of serotonergic transmission performed by serotonin autoreceptors (5-HT1A, 5-HT1B) localized in presynaptic neuron endings.

## 5. How to Correlate Extra-Synaptic Serotonin Diffusion Detected by Voltammetry and Serotonin Affinity for Membrane Lipids

How can we integrate the amphipathic characteristics of serotonin inherent to surface active compounds both into serotonergic neurotransmission mechanisms and in a more global vision of serotonin homeostasis? The clearing of neurotransmitters from the synaptic cleft is a critical step for neurotransmission because it is a discontinuous and iterative phenomenon. However, in the case of small amphiphilic neurotransmitters such as serotonin, the concept of specialized high fired synapse is less prevalent in view of the rather slow solubilization of serotonin in the synapse described in our model study. It depends on both of the serotonergic brain region and the predominance of functional type of serotonin receptors/transporters expressed [56]. Most serotonin receptors subtypes (5-HT1 to 5-HT7) are coupled to second messengers associated with paracrine neurotransmission, in contrast with 5-HT3 which is a ligand-gate ion channel associated with fast neurotransmission. Dahlström & Fuxe [57] have initially localized the serotonin pathway in the central nervous system of rat, concentrated in the cell bodies of the raphe nuclei of the brain stem. This observation was later confirmed in the human brain [58,59,60]. In human brain the highest levels of serotonin are present in the raphe nuclei, substantia nigra, striatum and hypothalamus [60]. In the substantia nigra pars reticula nucleus (SNr), the releasing sites are junctional with well characterized synapses [61]. On the other hand, in the dorsal raphe nucleus (DR) the situation seems more complex because it can be observed in cell-body regions with a predominance of non-junctional dendrites with also some axon releasing sites. However, the clearing of serotonin at the neuro-junctional and non-junctional spaces is chiefly performed by serotonin transporters [56]. Their targeting supposes in both cases the diffusion of serotonin along several micrometers, which is a significant distance from its releasing site. Studies carried out by fast scanned cyclic voltammetry using carbon-fiber microelectrodes, have measured serotonin concentrations of 0.1 µM and 0.055 µM, respectively, in the dorsal raphe (DR) and the SNr after one-pulse electrical stimulation which reasonably expected to come at least from the release of one vesicle of serotonin [62]. Given the tip diameter of the electrode (10–15 µm), this technique allows an overall real-time measurement of the serotonin concentration around the extra-synaptic space rather than in the synaptic volume. Under these experimental conditions, the possible interference of both transporter and/or auto-receptor dependent serotonin capture during the time necessary for serotonin diffusion has been minimized. In particular, the combined use of serotonin transport inhibitors as fluoxetine and the non-specific 5-HT1/5-HT2 antagonist (methiothepin) did not modify the response induced by one stimulus pulse. Moreover, a linear relationship has been established between the number of electric pulses elicited and the extracellular serotonin concentration detected. It is interesting to note a matching between the receptor and transporter affinities in the submicromolar range, and the serotonin concentrations recorded after one pulse stimulation. Overall, these results indicate that a correlation can be established between the serotonin efflux and the extra-synaptic location of these membrane proteins, consistent with a paracrine control of serotonergic neurotransmission [62]. In complement to these physiologically relevant observations, our previous experimental data highlight new aspects of serotoninergic neurotransmission. In synaptic vesicles, the concentration of serotonin is very high, in 250–400 mM range. For a nominal concentration of 250 mM and given the diameter of these vesicles (45–90 nm), it can be calculated that they contain between 7200 and 57,500 serotonin molecules [63]. These molecules are released in a space with the dimensions of SNr synapse (cylindrical approximation with 0.3 µm length and 0.15 µm width) [61,64].

Our experimental observations concerning the behavior of secreted serotonin suggests that a substantial fraction of vesicular serotonin remains insoluble within the synaptic cleft. Indeed, the limit of solubility of serotonin is 110 mM. Consequently, voltammetry measurements primarily reflect the soluble serotonin fraction that diffuses into the extrasynaptic space, estimated at approximately 44% of the vesicular content (about 3200 to 25,300 molecules, depending on vesicle diameter; Table 1). From these values, the synaptic concentration of soluble serotonin can be approximated as 7–52 mM in a cylindrical synapse. Assuming a varicosity density in the SNr of 9 × 10^6^ sites/mm^3^, each release site occupies a volume of 1.1 × 10^−16^ m^3^, which corresponds to at least one release site within a cube of 4.8 × 10^−6^ m edge length—smaller than the diameter of the microelectrode tip. Because extracellular serotonin levels depend on both tissue connectivity and cellular uptake, the effective diffusion volume was corrected by the extracellular volume fraction α (α = 0.2 for a small molecule such as serotonin) [65]. Under the additional assumption that sequestration by transporters and autoreceptors is negligible for the quantitative voltammetric estimate, the number of serotonin molecules released by a single pulse predicts an extracellular concentration of 0.25–1.90 µM (Table 1). The marked discrepancy between these calculated values and the experimentally recorded concentrations (0.05–0.10 µM) after a single pulse suggests the existence of an additional, previously unrecognized mechanism regulating extracellular serotonin, independent of uptake by transporters and/or activation of autoreceptors.

In these calculations, our estimates of peak 5-HT concentration (0.25–1.90 μM) rely on a simplified cylindrical geometry and instantaneous diffusion into a fixed extracellular volume fraction (α = 0.2), according to mathematical models [65,66,67]. We acknowledge this approach neglects several dynamic factors. In particular, real synapses exhibit complex geometries with tortuosity factors that impede diffusion, potentially creating localized concentration ‘hotspots’ significantly higher than our bulk estimates [68]. However, if realistic diffusion barriers and tortuosity are considered, the local retention of serotonin is enhanced, supporting our proposal of lipid-mediated buffering as a stabilizing mechanism alongside classical uptake.

## 6. Receptor-Independent Pathway for Serotonin Action and Serotonin Homeostasis in the Brain

These considerations lead to the view that voltammetrically measured serotonin could represent only a fraction of the total transmitter released into the extracellular space surrounding the release site. This interpretation relies on two key observations (13) together with our previous demonstration of the strong membrane affinity of serotonin. First, Bunin et al. [62] reported a linear increase in measured serotonin with the number of successive stimulus pulses, which suggests that the immediate target(s) of released serotonin are not saturable under these conditions. In light of our finding that neuronal membranes exhibit a high capacity to capture and retain serotonin through the combined action of gangliosides and cholesterol, it appears likely that, in physiological conditions, a major proportion of released serotonin could be rapidly reabsorbed by cell membranes within and beyond the synaptic cleft via a mechanism that does not require specific receptors or transporters.

Second, the combined application of a serotonin transporter blocker (fluoxetine) and a non-selective 5-HT1/5-HT2 antagonist (methiothepine) failed to alter the response to a single stimulus pulse [62]. This lack of effect indicates that, in the DR and SNr, serotonin release is not effectively buffered on the timescale of measurement by binding to transporters or receptors, but rather by the extensive “collecting funnel” surface provided by neuronal membranes in both synaptic and extrasynaptic compartments. In this framework, paracrine modulation of receptors and transporters by extrasynaptic serotonin remains a physiologically relevant mode of action, yet it likely accounts for only a partial control of serotonergic neurotransmission, which we estimate to involve approximately 2–15% of the soluble serotonin released in the SNr and 4–32% of the soluble serotonin released in the DR.

Serotonin is synthesized by a relatively small population of neurons, whose cell bodies are mainly located in the mesencephalon and rostral pons within the raphe nuclei. From these nuclei, ascending serotonergic projections reach forebrain structures via two principal pathways: a lateral pathway traveling through the internal capsule to innervate lateral cortical regions, and a medial pathway running in the medial forebrain bundle to innervate the hypothalamus, basal forebrain, and amygdala; the medial pathway also reaches medial cortical areas and the hippocampus through the cingulum bundle. In parallel, neurons in the lower pons and medulla give rise to descending serotonergic projections that target the brainstem and spinal cord.

Although serotonin is preferentially produced in the raphe nuclei, substantia nigra, striatum, and hypothalamus, lower levels are detected in the cerebellum and cerebral cortex, where it tends to concentrate in sensory and limbic areas. Thus, most regions of the central nervous system receive serotonergic input despite the relatively sparse number of classical synaptic contacts. Moreover, serotonergic neurons can release serotonin not only from axon terminals but also from soma, dendrites, and axonal varicosities at extrasynaptic sites, both by exocytosis [69] and via non-vesicular diffusion across neuronal membranes [70]. These features underscore the complexity of serotonergic neurotransmission and the broad, paracrine-like influence of serotonin, which is favored by its amphiphilic physicochemical properties.

**Figure 6:**
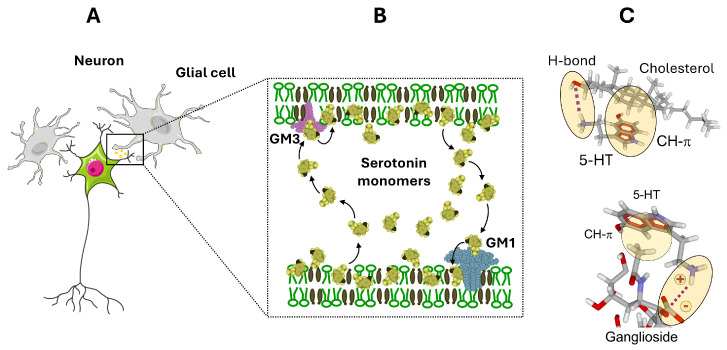
**Proposed model for the cholesterol/ganglioside-mediated shuttling of serotonin between neuronal and glial membranes.** Serotonin monomers in the extrasynaptic space partition into surrounding neuronal and glial cell membranes (**A**) by forming mixed cholesterol/serotonin complexes that insert into the lipid bilayer (**B**). In soma and dendrites of presynaptic neurons, these complexes accumulate in GM1-enriched lipid rafts, where CH–π and electrostatic interactions between serotonin and GM1 stabilize the amine at the membrane surface and modulate local 5-HT receptor activity. Cholesterol-serotonin interactions involve both CH–π and H-bond (**C**). In glial cells, serotonin associates with GM3-containing rafts (**B**), enabling uptake and transient storage of serotonin in membrane-embedded cholesterol/serotonin complexes, as illustrated in the left inset. This bidirectional exchange creates a dynamic cholesterol-dependent reservoir of serotonin distributed between neuronal and glial membranes. This model is based on the demonstrated affinity of serotonin for lipid raft components [27,43]. provided by Servier Medical Art (https://smart.servier.com), licensed under CC BY 4.0 (https://creativecommons.org/licenses/by/4.0/).

Serotonin’s distinctive ability to interact with both synaptic and extrasynaptic membranes leads to propose a revised view of serotonergic signaling. In this model, the post-exocytotic solubilizing action of gangliosides and cholesterol on serotonin aggregates, together with a constitutive pool of serotonin–cholesterol complexes, sustains long-lasting effects of serotonin that are partially uncoupled from classical vesicular release (Figure 6). In addition, because serotonin can be released at both synaptic and extrasynaptic sites, it may constitute a diffuse “broth” bathing neuronal membranes and surrounding microglia in regions of high serotonergic activity [69]. Consistent with synaptoneurolipidomic profiles [22], this membrane-inserted pool of serotonin, which modifies bilayer mechanical properties, has been proposed to contribute to a retro-control mechanism of brain excitability [71,72].

Serotonin’s impact on neuronal excitability can tentatively be linked to its direct association with membrane cholesterol. Molecular dynamics simulations and monolayer experiments suggest that cholesterol behaves as a privileged partner of serotonin in both liquid-disordered and liquid-ordered membrane phases, shifting the equilibrium between these two states. In this framework, serotonin–cholesterol complexes soften the stiffening effect of cholesterol in the disordered phase while attenuating its disordering effect in the ordered phase, thereby increasing interfacial tension and favoring the coalescence of nanoscale domains into larger membrane microdomains.

Such serotonin-dependent reorganization of membrane domains provides a plausible physicochemical basis for a feedback regulation of cerebral excitability operating through a “limited-invasion” percolation process [73], analogous to channeling an avalanche so that energy is dissipated without catastrophic spread. By locally raising excitability thresholds while still permitting signal propagation, serotonin may function as a kind of intrinsic “local anesthetic” that buffers the brain against overwhelming environmental inputs that could otherwise destabilize network activity. In this view, the persistent remodeling of neuronal membrane domains by membrane-inserted serotonin contributes to a foundational background state that absorbs a multitude of subthreshold stimuli and may underlie the basal electrical activity of the brain in standby mode, largely independent of the on–off firing dynamics that characterize conventional synaptic neurotransmission.

## 7. Correlation Between the Dysregulation of Serotonergic Activity and the Alteration of Cholesterol Metabolism in Neuronal Diseases

Such unexpected and ubiquitous properties of serotonin lead to the view that membrane lipids, particularly gangliosides and cholesterol, are key regulators of serotonergic neurotransmission and, more broadly, of brain serotonin homeostasis. Lipid-dependent synaptic and non-synaptic clearance of serotonin, together with extrasynaptic paracrine modulation of receptors and transporters, defines relatively slow processes that support a negative feedback control of brain activity and confer long-lasting effects distinct from those of fast glutamatergic or cholinergic synapses. The high capacity of neuronal membranes to retain serotonin, largely through gangliosides and cholesterol, is therefore likely to make a major contribution to the regulation of cerebral serotonin levels.

Disruption of brain serotonin homeostasis is known to alter multiple cognitive, behavioral, and developmental functions, and converging data implicate parallel disturbances of cholesterol metabolism in several neurological diseases (Table 2). Indeed, while these dysregulations are often attributed to protein-level defects (receptors/transporters), emerging evidence suggests that the lipid environment—and by extension, the “reservoir” capacity—plays a crucial background role in maintaining homeostatic signaling [12]. In Rett syndrome and Huntington disease, modeled, respectively, in Mecp2 knock-out mice [74] and zQ175 knock-in mice [75], abnormal cerebral cholesterol accumulation can be corrected by AAV-mediated overexpression of CYP46A1, which enhances cholesterol 24-hydroxylase activity [76], increases circulating 24(S)-hydroxycholesterol, and ameliorates disease-specific symptoms. Both disorders, in patients and animal models, also feature reduced brain serotonin turnover, reflected by decreased 5-HT synthesis and release in regions such as striatum, hippocampus, and frontal cortex, and lower 5-HIAA levels in cerebrospinal fluid [77,78,79,80,81]. Lipid raft–associated alterations in serotonergic signaling have also been implicated in Alzheimer’s disease [12,82], Parkinson’s disease [83], schizophrenia [84], and major depressive disorder [85].

The present work, together with earlier studies, suggests that serotonin binding to membrane cholesterol could be crucial for maintaining the homeostasis of both partners. Serotonin–cholesterol complexes alter membrane fluidity and lipid-domain organization, thereby modulating the function of serotonergic proteins (5-HT receptors and SERT) and likely the basal excitability of brain networks. When the neuronal cholesterol/serotonin balance is perturbed, as in the two genetic diseases discussed above, it remains an open but important question whether restoring cholesterol turnover with CYP46A1 gene therapy also normalizes brain serotonin levels and contributes to the clinical improvement observed.

More broadly, many mood and neuropsychiatric disorders, including major depression, bipolar disorder, obsessive–compulsive disorder, and schizophrenia, have been linked to disturbed cholesterol homeostasis (Table 2). Future longitudinal studies should therefore monitor brain serotonin status and, by extension, the membrane serotonin/cholesterol balance in these conditions. These considerations argue for a holistic medical approach that integrates somatic and mental health, with particular vigilance regarding potential mood effects in patients receiving lipid-lowering therapies such as statins [95], which can alter brain cholesterol dynamics and potentially destabilize the delicate cholesterol/serotonin equilibrium. In line with recent recommendations, a comprehensive understanding of serotonin biology and pharmacology should explicitly incorporate this membrane-mediated pathway into models of serotonergic signaling and into the design and monitoring of therapeutic interventions.

## 8. How Fundamental Parameters of Biology Are Integrated in the New Model of Serotonergic Transmission

**Figure 7:**
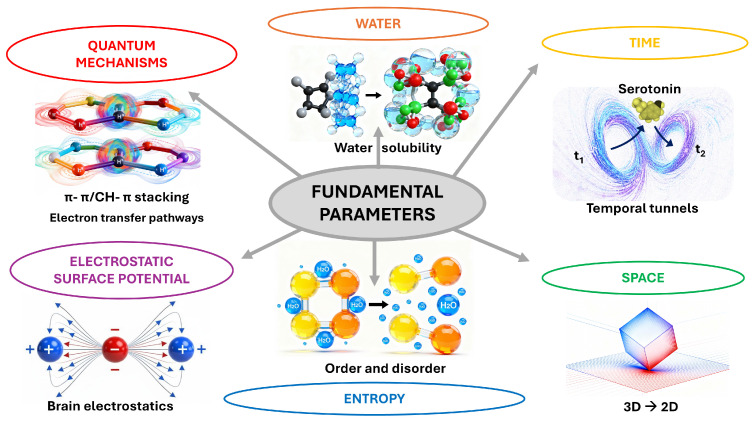
**Fundamental parameters governing neuromodulator–membrane interactions at the serotonergic synapse.** The central scheme highlights six interconnected levels of description: quantum mechanisms, electrostatic surface potential, water, entropy, and spatiotemporal organization. Quantum-scale π–π and CH–π stacking interactions control the recognition of aromatic neurotransmitters such as serotonin by lipids and proteins. Serotonin can also create structured systems with enhanced electron transfer pathways, showing that quantum electronic properties extend beyond simple non-covalent interactions to influence supramolecular organization. Electrostatic fields in the brain shape long-range forces that guide ligand trajectories at charged membrane interfaces. Water and entropy determine solubility, phase separation, and the ordered or disordered states of membrane-associated molecular assemblies, thereby tuning receptor accessibility. Finally, time and space are represented by serotonin moving through “temporal tunnels” (t_1_ for serotonin aggregation in synaptic vesicles during exocytosis, t_2_ for serotonin insertion in brain cell membranes) and by 3D-to-2D transitions at membrane surfaces, emphasizing that neuromodulator signaling emerges from coordinated quantum, thermodynamic, electrostatic, and geometric constraints.

Our model integrates six fundamental parameters—water, time, space, entropy, electrostatic surface potential, and quantum mechanisms—[96] that together define the physical basis of serotonergic transmission (Figure 7). Water provides the medium for solvation and molecular mobility. Time structures the sequence of events across successive kinetic domains. Space constrains diffusion and guides molecular encounters at the membrane interface. Entropy drives the transition from ordered aggregates to freely dispersed serotonin molecules. Electrostatic potential governs the directed migration of serotonin toward negatively charged ganglioside domains. Finally, serotonin exhibits several quantum mechanical properties that are relevant to both its molecular structure and potential biological functions. The indole ring system displays characteristic quantum behaviors related to electron delocalization, orbital interactions, and potential quantum coherence effects [97]. These quantum mechanisms operate at the level of non-covalent π–π [98] and CH–π interactions [99], ensuring molecular specificity and reversible binding. These parameters act in concert to couple thermodynamic and structural processes within a unified biophysical framework. Serotonergic transmission thus appears as an emergent property of interacting physical fields, through which energy dissipation and molecular organization are continuously balanced to sustain biological communication.

Concerning time, our model identifies two potential limiting steps—or “temporal tunnels”—in serotonergic transmission. The first arises immediately after exocytosis and corresponds to the time required for serotonin aggregates to dissociate in the extracellular environment. This process depends on the interplay between aggregate stability, solvation forces, and progressive binding of serotonin molecules to gangliosides at the membrane surface. In an in vitro model of ganglioside-induced serotonin solubilization, the temporal scale was estimated to several minutes [43]. The second temporal tunnel begins once serotonin inserts into the plasma membrane of brain cells—both neurons and glial cells. Biophysical studies, including solid-state NMR spectroscopy, have confirmed that serotonin binds continuously to the membrane interface, where it modulates lipid order and membrane mechanics [55]. These experimental data suggest the interesting possibility that serotonin could diffuse laterally within the lipid bilayer, allowing its local accumulation, long-term stabilization, and eventual release toward specific receptor microdomains. This membrane-associated reservoir could provide a mechanism for serotonin storage and delivery that operates independently of synaptic vesicle exocytosis, thereby extending the temporal scope of serotonergic signaling far beyond the millisecond scale of conventional synaptic events. In our model, this previously neglected aspect of serotonin action could easily occur on a temporal scale of several hours, and perhaps days.

The entropic relaxation that drives aggregate dissolution also defines the temporal scale of this first tunnel. In effect, time emerges as the phenomenological expression of entropy production: the more rapid disorder increases through the dispersal of serotonin molecules and the liberation of structured water, the shorter the duration of this tunnel. Conversely, if aggregate stability or local solvation constraints delay the entropic expansion of the system, the temporal tunnel is prolonged. Thus, entropy and time are probably intimately coupled parameters within serotonergic dynamics, the former governing the direction of molecular evolution and the latter quantifying its spatial distribution.

The interplay between time, water and entropy thus represents a fundamental axis of our model. Entropy determines the degree of molecular and solvent reorganization, while time reflects the kinetic unfolding of these entropic processes. In serotonergic transmission, the first temporal tunnel captures this coupling most clearly: as entropy increases through the dissolution of aggregates and the restoration of water dynamics, time manifests as the measurable duration of this transformation. Both parameters therefore describe complementary aspects of the same underlying phenomenon—the progressive relaxation of an initially ordered molecular state toward equilibrium. In this sense, the dynamics of serotonin provide a microcosm of the broader principle by which biological systems translate thermodynamic irreversibility into temporal order.

The relaxation process that follows the entropic phase may give rise to a new form of organization governed by electrostatic surface potential. As individual serotonin molecules become solvated and free to interact, their migration toward ganglioside-rich membrane domains can be guided by the local electrostatic landscape. The negatively charged sialic acid residues of gangliosides generate an electronegative field that attracts protonated serotonin, re-establishing order after the entropic dispersion of the aggregates. This transition illustrates the functional continuity between parameters: entropy sets serotonin molecules in motion, while electrostatic surface potential directs this motion toward structured membrane domains where signal transmission is reinitiated. Entropy thus disperses, and electrostatics reorders—the two forming a complementary cycle that underpins the dynamic coherence of serotonergic signaling.

At the deepest level of organization, the electrostatic alignment of serotonin and its membrane partners may prepare the stage for quantum mechanisms to operate. Once serotonin molecules approach ganglioside and cholesterol microdomains, their interactions become dominated by short-range quantum forces, notably π–π stacking and CH–π interactions. These subtle resonant couplings bridge molecular and electronic structures, stabilizing transient complexes and facilitating selective energy transfer within the membrane environment. In particular, π–π interactions maintain the coherence of serotonin aggregates before dissociation, while CH–π contacts between serotonin, cholesterol, and GM1 contribute to their membrane anchoring and dynamic exchange with receptors. Quantum properties may also explain some key physicochemical characteristics of serotonin, e.g., its exceptionally high membrane partitioning coefficient (~1200 in mole fraction units) [100]. This strong membrane affinity relies on specific quantum-mediated interactions: the protonated primary amine forms a salt bridge with lipid phosphate groups, anchoring serotonin at the membrane interface with its aromatic ring system pointing inward toward the hydrophobic core. The electron-rich indole π-system resides preferentially between the phosphate and carbonyl groups of lipids, where π-orbital interactions with the membrane’s electronic structure stabilize the association. Recent work also showed that serotonin can modulate proteinoid self-assembly by creating structured systems with enhanced electron transfer pathways, demonstrating how quantum electronic properties extend beyond simple molecular interactions to influence supramolecular organization [101]. The amphiphilic nature of serotonin—combining a charged ammonium group with an aromatic hydrophobic ring—creates a molecule capable of both hydrophilic salt-bridge formation and hydrophobic π-stacking, making its aggregation and partitioning highly sensitive to local electronic environments and membrane composition [102].

Hence, we propose that quantum mechanisms provide the fine-tuning needed to translate electrostatic attraction into molecular specificity and signal precision. In our model, electrostatics define the spatial trajectory of serotonin, and quantum effects govern the intimacy and reversibility of its binding events—embodying the final link in the chain that connects energy dissipation to information flow.

Together, the successive examination of these parameters reveals a coherent physical logic underlying serotonergic transmission, which can now be summarized within a unified conceptual framework (Figure 7). By grounding neuronal communication in these universal physical parameters, the present framework extends the definition of serotonergic transmission beyond its biochemical boundaries, suggesting that the principles governing life and cognition may ultimately converge within a common physicochemical language. Our study further suggests that brain cells may be continuously exposed to serotonin over time scales substantially longer than currently assumed. Such prolonged exposure, enabled by the insertion of serotonin into neuronal and glial membranes, allows local storage and gradual release that extend beyond classical synaptic transmission. This observation calls for a re-evaluation of current pharmacological models of serotonergic regulation. In particular, it may prompt a reassessment of the mechanisms and long-term efficacy of antidepressant drugs such as fluoxetine, which predominantly act on synaptic rather than membrane-associated serotonin dynamics. As also suggested by Dey et al. [55], a membrane-mediated pathway of serotonin, independent of conventional receptor-mediated signaling, allows for direct modulation of cellular functions through altered membrane mechanics. This mechanism could become particularly critical when extracellular serotonin is maintained at elevated levels, such as during treatment with serotonin transporter blockers. Viewed through the lens of the fundamental parameters, this reinterpretation of serotonin dynamics reinforces the idea that therapeutic modulation of mood and cognition ultimately depends on the interplay between physical constraints, temporal organization, and quantum-level interactions that together define the living state.

## 9. Alternative Models

Current understanding of serotonergic transmission is dominated by two primary frameworks: the “wiring transmission” model, where rapid reuptake by the serotonin transporter (SERT) restricts signals to the synaptic cleft [103], and the “volume transmission” model, where serotonin diffuses widely to reach distant extrasynaptic receptors [104,105]. While these frameworks explain bulk kinetics [106], they largely treat the plasma membrane as an inert boundary. Consequently, slow clearance is often attributed solely to physical diffusion barriers [107], such as glial sheath geometry or extracellular matrix viscosity. However, these models struggle to account for the modulation of signaling observed when membrane lipid composition is altered (e.g., in depression or aging) in the absence of significant changes in SERT expression. Our “lipid raft reservoir” model addresses this gap. It introduces a lipid-dependent buffering capacity that complements canonical clearance mechanisms [108]. By sequestering serotonin within membrane microdomains, this reservoir may modulate the “free” pool available for reuptake and diffusion, thereby linking membrane lipid homeostasis directly to synaptic efficacy—a layer of regulation distinct from both SERT kinetics and physical diffusion barriers. This framework aligns with emerging views on membrane lipid composition and raft-localized signaling influencing 5-HT receptor function and transporter interactions [109,110], suggesting new parameters for modeling clearance that incorporate lipid-phase occupancy, affinity-based sequestration, and dynamic raft remodeling.

Several observations suggest that serotonin can be released from serotonergic neurons outside of synapses both by exocytosis [69] and by simple non-vesicular diffusion through a concentration gradient across the plasma membrane surrounding the soma, dendrites, and axonal varicosities [70]. This process of releasing serotonin continuously with low noise outside the synapses allows it to soak up membranes of cells located around the emission sites. It may represent a non-wiring mode of serotonergic transmission associated with a volume transmission model which might function in parallel with the wiring transmission mode.

Finally, while our model focuses on the modulation of lipid-raft reservoirs, we recognize that the therapeutic latency and efficacy of selective serotonin reuptake inhibitors (SSRIs) involve multiple, non-mutually exclusive mechanisms. (i) It is known that antidepressants accumulate in lipid rafts and displace G proteins to non-raft domains, thereby enhancing cAMP signaling [111]. This mechanism operates via protein-lipid interface modification rather than direct serotonin sequestration. We hypothesize that these same lipid-drug interactions may collaterally displace serotonin from ganglioside binding sites, but we distinguish this as a theoretical extension of the established G-protein effect. (ii) as discussed above, SERT-independent transport mechanisms facilitate low-affinity serotonin clearance that is insensitive to SSRIs [112]. These pathways may buffer extracellular serotonin when SERT is blocked, offering a kinetic explanation for incomplete SSRI efficacy that is distinct from our reservoir concept. (iii) Beyond specific raft interactions, amphiphilic antidepressants can alter bulk bilayer properties (elasticity, curvature), which nonspecifically modulates the function of embedded proteins like channels and receptors [113].

## 10. Limitations and Perspectives

The present model assumes that a fraction of extracellular serotonin may exist in an aggregated (or otherwise sequestered) state; however, direct quantification of serotonin aggregation in vivo in the synaptic cleft is currently lacking. Our parameterization necessarily relies on data obtained in simplified systems (e.g., aqueous solutions and model membranes), which cannot reproduce key determinants of the synaptic milieu (local geometry, macromolecular crowding, extracellular matrix composition, lipid/protein surfaces, ionic strength, and rapid clearance by transporters). Accordingly, the magnitude of any “aggregated fraction” should be viewed as an upper-bound/working estimate rather than a physiological constant, and the model’s main value is to highlight testable predictions (conditions under which aggregation/sequestration would become non-negligible and expected kinetic consequences). In this context, receptor-independent membrane effects reported in cell/model-membrane settings [43,71,100] support the plausibility of membrane-associated serotonin pools, but do not by themselves establish their prevalence in vivo.

It is also important to emphasize that the divergence between our calculated serotonin concentrations and available voltammetry measurements does not, in itself, constitute definitive proof of a serotonin reservoir or aggregation mechanism. Rather, this quantitative discrepancy serves as a hypothesis-generating observation—alongside potential factors like microdomain-restricted diffusion or rapid local uptake—that warrants further investigation. Therefore, our model should be viewed as a theoretical framework that tests the plausibility of the reservoir hypothesis, rather than as a demonstration of its existence.

Finally, to further elucidate the physical nature of the serotonin–membrane interaction and discriminate between classical electrostatic forces and potential quantum-level contributions (e.g., vibrational coupling), we propose comparative binding studies using deuterated serotonin. Since isotopic substitution alters molecular vibrational modes and tunneling probabilities without modifying the steric or electrostatic profile [114], a significant difference in binding kinetics would provide experimental support for non-classical mechanisms [115].

## Figures and Tables

**Table 1:** **Estimated serotonin content and synaptic release parameters for small and large vesicles.** Vesicle diameters and corresponding intravesicular volumes were used to estimate the total number of serotonin (5-HT) molecules at a nominal concentration of 250 mM. The soluble fraction available for immediate release was limited to ≤110 mM, reflecting partial intravesicular aggregation due to the limit of solubility of serotonin. The resulting peak 5-HT concentration in the synaptic cleft was inferred from diffusion models assuming instantaneous exocytosis in a typical cleft. Larger vesicles store and release greater amounts of serotonin, leading to proportionally higher transient concentrations at the receptor interface. Detailed calculations are provided in the Supplementary Materials.

Vesicle Type	Diameter	Volume (L)	Total 5-HT (250 mM)	Soluble 5-HT (≤110 mM)	Peak 5-HT in Cleft
**Small**	45 nm	4.77 × 10^−20^	~7200 molecules	~3200 molecules	~0.25 µM
**Large**	90 nm	3.82 × 10^−19^	~57,500 molecules	~25,300 molecules	~1.90 µM

**Table 2:** **Dysregulation of serotonergic activity in neuronal diseases: connection to membrane/lipid rafts.**

Disease/Disorder	Serotonergic Dysregulation Mechanism	Connection to Membrane/Lipid Rafts
Rett syndrome [74]	Decreased serotonin synthesis and release [86]	Abnormal cerebral cholesterol accumulation [87]
Huntington disease [75]	Decreased serotonin synthesis and release [88]	Abnormal cerebral cholesterol accumulation [76]
Alzheimer’s disease [12]	Reduced cortical serotonin levels [89]	Ganglioside/cholesterol regulation of 5-HT1A receptor [16] and neurotoxic oligomer formation by Aβ protein [90]
Parkinson’s disease [83]	Degeneration of serotonergic terminals contributes to motor and non-motor symptoms [91]	Ganglioside/cholesterol regulation of 5-HT1A receptor and neurotoxic oligomer formation by α-synuclein [92]
Schizophrenia [84]	Imbalance in cortical 5-HT2A (hyperactivity) vs. 5-HT1A signaling [93]	Altered brain lipid composition affects 5-HT2A receptor [12]
Major depressive disorder [85]	Alterations in 5-HT receptor sensitivity [94]	Altered lipid microenvironments can destabilize SERT and 5-HT1A receptors [47]

## Data Availability

The raw data supporting the conclusions of this article will be made available by the authors on request.

## Supplementary Materials

The following supporting information can be downloaded at: https://www.mdpi.com/article/10.3390/life16010118/s1, Calculations for Synaptic Vesicle of serotonin Content and Release.
