# Supplementary material for: Serotonergic Signaling Rewired: A Lipid Raft-Controlled Model of Synaptic Transmission Grounded in the Fundamental Parameters of Biological Systems
Source: Life (Basel). 2026 Jan 13;16(1):118. doi: 10.3390/life16010118 (PMC12842897; doi:10.3390/life16010118)
Supplement: Supplementary file 1 [file life-16-00118-s001.zip › life-4083356-supplementary.pdf]

Supplementary Materials

Serotonergic signaling rewired: a lipid raft-controlled model of synaptic transmission grounded in the fundamental parameters of biological systems

Jacques Fantini [1*], Marine Lefebvre[2,3], Nouara Yahi[1,] and Henri Chahinian[1]

[1] Department of Biology, Faculty of Medicine, University of Aix-Marseille, INSERM UA16, 13015 Marseille, France
[2] IHU Méditerranée Infection, 19-21 Boulevard Jean Moulin, 13005 Marseille, France
[3] Microbes Evolution Phylogeny and Infections (MEPHI), Aix-Marseille Université, 27 Boulevard Jean Moulin, 13005 Marseille, France
* Correspondence: jacques.fantini@univ-amu.fr

Calculations for Synaptic Vesicle of serotonin Content and Release (data summarized in Table 1).

Global calculation for a synaptic vesicle with a diameter of 90 nm.

Assumptions:
The model assumes a spherical vesicle, a total vesicular concentration of 250 mM, and a solubility limit of 110 mM. The extra-synaptic space is modeled as a cube with an edge length of 4.8 µm, with an available volume factor of 0.2 (representing porosity/tortuosity).

Calculations are based on standard mathematical formulas. Final results are rounded for readability while maintaining reasonable precision.

1. Vesicle Volume

Data:

- Diameter = 90 nm →Radius $r = 45\text{nm}$.

Formula:

- Volume of a sphere: $V = \frac{4}{3}\pi r^3$

Calculation:

$$r^3 = 45^3 = 91{,}125 \text{ nm}^3$$
$$V = \frac{4}{3} \times \pi \times 91{,}125 \approx 1.333 \times 3.1416 \times 91{,}125 \approx 381{,}700 \text{ nm}^3$$

Conversion:

- To cubic meters ($1\ \text{nm}^3 = 10^{-27}\ \text{m}^3$):
$$V \approx 381{,}700 \times 10^{-27} = 3.82 \times 10^{-22}\ \text{m}^3$$
- To liters ($1\ \text{m}^3 = 1{,}000\ \text{L}$):
$$V \approx 3.82 \times 10^{-2}\ \times 1{,}000 = 3.82 \times 10^{-1}\ \ \text{L}$$

Result: The vesicle volume is approximately $382{,}000\ \text{nm}^3$, corresponding to $3.82 \times 10^{-22}\ \text{m}^3$or 3.82×10^{-19} L.

2. Number of Serotonin Molecules at Total Concentration (250 mM)

Data:

- Concentration $C_{total} = 250\ \text{mM} = 0.25\ \text{mol/L}$
- Volume $V = 3.82 \times 10^{-19}$ L
- Avogadro constant $N_A = 6.022 \times 10^{23}$ molecules/mol

Formula:

- $N = C \times V \times N_A$

Calculation:

$$n(\text{moles}) = 0.25 \times 3.82 \times 10^{-19} \approx 9.55 \times 10^{-20}\ \text{mol}$$
$$N(\text{molecules}) = 9.55 \times 10^{-20} \times 6.022 \times 10^{23} \approx 57{,}500\ \text{molecules}$$

Result: Approximately **57,500 molecules** of serotonin in total (at 250 mM).

3. Number of Soluble Molecules (at Solubility Limit of 110 mM)

Data:

- Solubility limit $C_{sol} = 110\ \text{mM} = 0.11\ \text{mol/L}$(excess forms aggregates).

Calculation:

$$n_{sol} = 0.11 \times 3.82 \times 10^{-19} \approx 4.20 \times 10^{-20}\ \text{mol}$$
$$N_{sol} = 4.20 \times 10^{-20} \times 6.022 \times 10^{23} \approx 25{,}300\ \text{molecules}$$

Result: Approximately **25,300 molecules** of serotonin in soluble form.
(Interpretation: At a total concentration of 250 mM, only ~44% is soluble; the remainder is stored in a bound or dense form (aggregates), consistent with real vesicles.)

4. Concentration in the Extra-synaptic Space (Uncorrected)

Data:

- Released molecules = 25,300 (soluble fraction).
- Synaptic cube edge $a = 4.8\ \mu\text{m} = 4.8 \times 10^{-6}$ m.

Formula:

- Volume of cube $V_{syn} = a^3$

Calculation:

$$a^3 = (4.8 \times 10^{-6})^3 = 1.10592 \times 10^{-16}\ \mathrm{m}^3$$

- Converted to Liters:

$$V_{syn(L)} \approx 1.10592 \times 10^{-1}\ \ \times 1{,}000 = 1.11 \times 10^{-1}\ \ \mathrm{L}$$

- Concentration calculation:

$$n_{released} \approx 4.20 \times 10^{-20}\ \mathrm{mol}$$

$$C_{syn} = \frac{4.20 \times 10^{-20}}{1.11 \times 10^{-13}} \approx 3.79 \times 10^{-7}\ \mathrm{mol/L} = 379\ \mathrm{nM}$$

Result: Immediate concentration ≈**380 nM**.

5. Concentration Corrected for Available Volume Factor (0.2)

Data:

- Accessible volume is 20% of the geometric volume.
- Factor $f = 0.2$.

Calculation:

$$V_{accessible} = V_{syn(L)} \times 0.2 = 1.11 \times 10^{-1}\ \ \times 0.2 = 2.22 \times 10^{-14}\ \mathrm{L}$$

$$C_{corrected} = \frac{4.20 \times 10^{-20}}{2.22 \times 10^{-14}} \approx 1.89 \times 10^{-6}\ \mathrm{mol/L} = 1{,}890\ \mathrm{nM}$$

Result: Corrected concentration ≈**1,900 nM (1.9 μM)**.
